# An unusual finding in a desmoid-type fibromatosis of the pancreas: a case report and review of the literature

**DOI:** 10.1186/s13256-018-1635-x

**Published:** 2018-05-12

**Authors:** Joseph Clarence Torres, Chen Xin

**Affiliations:** Department of Endocrine and Breast Surgery, First Affiliated Hospital of Chongqing Medical University, No.1 Yixueyuan Rd, Yuzhong District, Chongqing, 400016 People’s Republic of China

**Keywords:** Desmoid-type fibromatosis, Aggressive fibromatosis, Pancreatic desmoid tumor, β-catenin, S100 protein, Radical resection

## Abstract

**Background:**

Desmoid-type fibromatoses are rare benign and fibrous tumors that account for approximately 0.03% of total neoplasms. Within this category of neoplasms, pancreatic desmoid-type fibromatosis is an extremely rare subgroup, accounting for approximately 5% of desmoid-type fibromatoses. Although the etiology is unknown, some risk factors include trauma, surgery, family history of desmoid tumor, pregnancy, use of contraceptives, genetic mutation, and familial adenomatous polyposis or Gardner syndrome. Desmoid-type tumors are primarily diagnosed by pathological and immunohistochemical studies. The treatment of choice is surgical resection of the tumor. Systemic chemotherapy and radiotherapy are optional treatment approaches for patients with high risk for surgery.

The following is a report of an unusual case of a pancreatic desmoid-type fibromatosis in which a very rare variation in the immunohistochemistry findings was demonstrated: slides were immunopositive for S100 protein and not immunonegative. Most desmoid tumors are immunonegative for S100 protein with just a few cases being positive for this protein.

**Case presentation:**

We describe the case of a 15-year-old boy, a Chinese national, who was diagnosed as having a pancreatic desmoid-type fibromatosis. He was a healthy individual who was incidentally diagnosed with an abdominal mass. His chief complaints were mild generalized abdominal pain for 1 week, nausea, vomiting, and a low-grade fever. An enhanced computed tomography scan of his abdomen showed a large cystic mass in the anterior surface of the body of his pancreas. He underwent a radical resection of the pancreatic mass, partial pancreatectomy, splenectomy, segmental resection of transverse colon and distal jejunum, and subsequently a one-stage jejunojejunostomy and colonic anastomosis. Postoperatively, he was diagnosed as having a desmoid-type fibromatosis of the pancreas by pathological and immunohistochemical studies.

**Conclusions:**

The diagnosis of desmoid-type fibromatosis is usually incidental and challenging. Pathological and immunohistochemical testing are essential for confirming diagnosis where demonstration of β-catenin nuclear staining is probably the single most important characteristic. Other markers, such as vimentin, are usually positive, while S100 protein is usually negative. Our case however, confirms that there are rare cases that can be S100 positive. This is the first diagnosed case of pancreatic desmoid-type fibromatosis with S100 protein positivity.

## Background

Desmoid-type fibromatosis or aggressive fibromatosis is also known as a desmoid tumor (DT). It is a rare group of benign neoplasms that result from the proliferation of well-differentiated mesenchymal fibroblasts. These tumors are known to be locally invasive with no evidence of distant metastatic potential. The advanced stages of the disease, however, can still be life-threatening due to local tissue invasion [1, 2]. Although DTs are generally located extra- abdominally (abdominal wall) or intra-abdominally, they can be found in any fibrous connective tissues throughout the body. They can present as solid, cystic, or solid-cystic masses. The etiology of these tumors is not well defined, although there is a tendency for them to occur sporadically or in association with familial adenomatous polyposis (FAP) in approximately 10 to 20% of cases**.** The risk factors are history of trauma, irradiation, positive family history for DT, surgery, pregnancy, use of contraceptives, genetic mutation, and FAP or Gardner syndrome [1–4]. The genetic mutation that is thought to be involved in DTs associated with FAP is a somatic mutation in the 3' region of the adenomatous polyposis coli (*APC*) gene or β-catenin (*CTNNB1*) gene. The protein APC regulates cellular β-catenin which is involved in wound healing and fibroprofileration. APC and β-catenin are both members of the Wnt signaling pathway, and both can be aberrantly expressed in DTs irrespective of familial or sporadic origin. The consequence of this is a translocation of cytoplasmic β-catenin into the nucleus that activates the expression of T-cell factor and this upregulates the expression of downstream genes such as cytochrome c oxidase 2 (*COX2*), platelet-derived growth factor (*PDGF*), matrix metallopeptidase (*MMP*), hyaluronan-mediated motility receptor (*RHAMM*), and retinoblastoma 1 (*RB1*) [5–7].

The following is an unusual case of an isolated and sporadic desmoid-type fibromatosis located in the body and tail of the pancreas. In this case we discovered a rare immunohistochemistry presentation of a pancreatic DT; therefore, our objective is to highlight the current pathological and immunohistochemical diagnostic criteria of this entity while demonstrating this rare finding. This is the first case of a pancreatic DT, diagnosed on 12 January 2017, at the First Affiliated Hospital of Chongqing Medical University, Chongqing, People’s Republic of China. We also present an updated literature review on desmoid-type fibromatosis of the pancreas.

## Case presentation

Our patient is a 15-year-old boy, a Chinese national, with no significant medical history. His personal and family history were both negative for FAP and Gardner syndrome. He had a normal infancy and early childhood and was fully vaccinated. Significantly, he has a family history of pulmonary tuberculosis, hemophilia, and he is an offspring of parents who are first-generation cousins. He was referred to our institution after visiting his local hospital for a routine physical examination where he was diagnosed as having an upper abdominal mass. A computed tomography (CT) scan at his local hospital revealed an abdominal cystic mass adjacent to his pancreas measuring 11.5 × 9.0 cm.

**Fig. 1:**
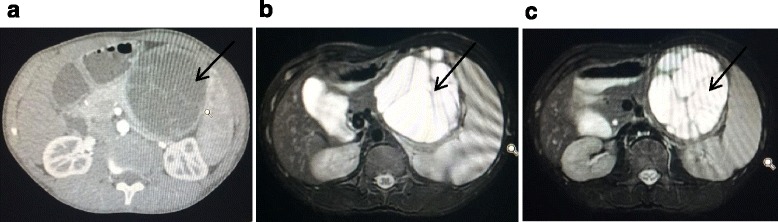
**a** Enhanced computed tomography scan images of abdomen without contrast of cystic mass at body and tail of pancreas. **b** and **c** Enhanced computed tomography scan of abdomen with contrast of abdomen both showing mass adjacent to body and tail of pancreas indicated by *arrows*

He was initially seen at our Surgical Out-patient Department. His chief complaints were a 1-week history of mild abdominal pain (mainly in the epigastric region), nausea, vomiting, and a low-grade fever. On physical examination his abdomen was slightly distended, with dullness on percussion, and generalized tenderness on palpation. A large mass measuring approximately 7 × 10 cm was palpable in his left upper abdomen extending from the epigastric to left hypochondriac region; it was of a hard consistency with well-defined surface and borders and poor mobility. In our hospital, an enhanced CT scan of his abdomen was done and this revealed a large cystic mass in the anterior aspect of the body of his pancreas measuring 9.3 × 8.5 cm with characteristic of pancreatic cancer (of pancreatic origin). Other radiological differentials were a solid pseudopapillary tumor or cystadenoma. A large splenomegaly with compression of splenic vein was also noted. He was subsequently admitted for further investigation and management (Fig. 1).

An abdominal magnetic resonance imaging (MRI) was also done and this confirmed a huge cystic mass measuring approximately 8.1 × 9.3 × 11cm closely adherent to the body and tail of his pancreas. Our patient was scheduled for an elective exploratory laparotomy with a preoperative diagnosis of pancreatic cancer of unknown origin. Intraoperatively, a decision was made to perform a radical resection of the pancreatic mass. Due to the large size of the tumor and extensive local adhesions encountered, a partial pancreatectomy, total splenectomy, segmental resection of transverse colon and distal jejunum, and subsequently a one-stage jejunojejunostomy and colonic anastomosis were also performed.

Preoperative laboratory investigations (complete blood count, liver function test, renal function test, electrolytes) were all unremarkable. Intraoperative findings were a grossly enlarged spleen measuring approximately 13 × 9 cm and a mass approximately 20 × 14 cm at the body and tail of his pancreas with adhesion to the posterior wall of his stomach, transverse colon, and distal portion of jejunum. There were also multiple enlarged lymph nodes. The duration of the surgery was 5 hours and 5 minutes and it was completed without any incidents. There was a total blood loss of 1700 ml; therefore, 700 ml of packed red blood cells and 400 ml of plasma were transfused intraoperatively. Postoperatively, our patient progressed satisfactorily and was discharged from hospital in a clinically improved and stable condition. He was seen in follow-up clinic 2-months post-operation with a repeat abdominal CT scan which revealed no evidence of recurrence of the abdominal mass. He is currently not being considered for adjuvant chemotherapy or radiotherapy but will continue to attend out-patient clinics for close monitoring and care.

Pathology and immunohistochemistry studies of his pancreas, jejunum, and spleen showed transitional hyperplasia and spindle cells atypia with invasion of his pancreas and muscular layer of his intestinal wall. There was evidence of reactive hyperplasia of lymph nodes and mild congestion of his spleen.

Individual immunohistochemistry results were β-catenin (+), vimentin (+), S100 (+), Ki67 < 1% (+), DOG1 (−), CD117 (−), smooth muscle actin (SMA) (−), desmin (−), ALK (−), CD34 (−), calretinin (−), WT1 (−), CK5/6 (−), EMA (−), D2-40 (−), and MC (−). The combined pathology and immunohistochemistry results were consistent with aggressive fibromatosis or desmoid-type fibromatosis of the pancreas.

## Discussion

Desmoid-type fibromatosis in general, has an annual incidence of approximately 2 to 5 cases per 1 million in USA and European populations and accounts for approximately 0.03% of all tumors and 3% of fibrous tumors [8–10]. Although DTs can occur at any age between the ages of 15 and 60 years, these tumors occur frequently during early adolescence and around 30 years [5, 11]. In the pediatric population, DTs are located primarily in extra-abdominal structures, affecting mainly the skeleton, skeletal muscle, adjacent fascia, aponeurosis, and periosteum. In the adult population, however, they are frequently located intra-abdominally, affecting most commonly the gastrointestinal and genitourinary tracts. Intra-abdominal DTs represent approximately 8% of total DTs [12] and are primarily of mesenteric connective tissues or retroperitoneum. They are rarely symptomatic and therefore their diagnosis is generally incidental.

The above-mentioned case of pancreatic DT is to the best of our knowledge the 27th case documented in the English literature since 1980. This is the fourth documented case in English in the People’s Republic of China [5, 13, 14]. The 26 previously documented cases can be seen in the references below [1, 2, 4, 5, 10, 13–18]. DT of pancreatic origin is an extremely rare subset of DTs [4].

The clinical presentation of DTs is usually asymptomatic or they can present with nonspecific signs and symptoms. These symptoms are usually related to the location of growth of the tumor and invasion of adjacent structures. Intra-abdominal DT can present nonspecific abdominal discomfort or pain and weight loss. If located in the gastrointestinal tract or urinary tract, symptoms of compression or obstruction may be the predominant clinical manifestations. DT of pancreatic origin is similar to pancreatic cancer and therefore usually silent, but occasionally DT of pancreatic origin and pancreatic cancer can cause epigastric pain that seldom radiates to the back. A painless jaundice can be seen if DT is located in the head of the pancreas. Weight loss is, however, often seen in pancreatic DT due to chronic aversion to food [1, 5]. In our case, our patient referred mild abdominal pain, nausea, vomiting, and a low-grade fever.

Possible complications are also determined by the location of the tumor. For instance, a tumor located in the extremities can result in neuropathic pain and debilitating sequelae. Intra-abdominal DTs can result in intestinal obstruction and fistulation, ischemia, hemorrhage, and perforation or ureteric obstruction or recurrence [19].

A clinical diagnosis of pancreatic DT is almost impossible due to its nonspecific clinical presentation. It is always essential to commence with a thorough clinical history and physical examination. The clinical history must specify if there is personal and/or family history of genetic diseases. CT scans and MRI are valuable diagnostic tools. Cross-sectional imaging of the affected area with CT or MRI is needed to define the relationship of the tumor to adjacent structures in order to assess resectability. The definitive diagnosis of DT however, is confirmed by pathological and immunohistochemical findings [1, 4, 5].

**Fig. 2:**
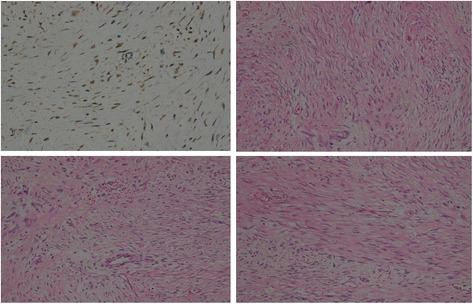
Immunopositivity for nuclear staining of β-catenin (40 × 10 microscopic resolution)

**Fig. 3:**
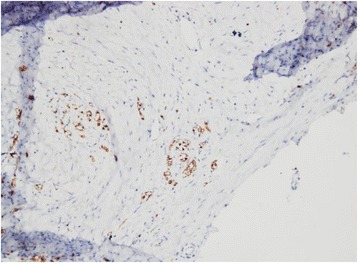
S100 protein immunopositivity (40 × 10 microscopic resolution)

Although not pathognomonic to DTs, spindle cells are common histopathological findings. Immunohistochemistry findings for intra-abdominal DT are: vimentin (+) and β-catenin (+) and immunonegative for SMA such as S100, CD117, and CD34 [5]. Our patient’s results were consistent with these criteria with the exception of S100 positivity (Figs. 2 and 3).

**Fig. 4:**
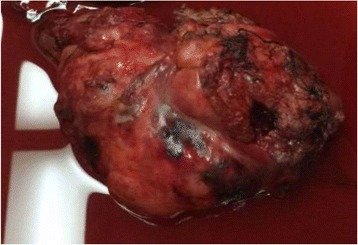
Gross sample of pancreatic desmoid-type tumor measuring approximately 20 × 14 cm

In general, desmoid-type fibromatoses are usually immunonegative for S100 protein, although a few rare cells can present S100 immunopositive slides [20]. In the presence of this, demonstration of nuclear β-catenin staining is essential for diagnosis. Our definitive diagnosis was confirmed by positive β-catenin nuclear staining as clearly illustrated by Figs. 3 and 4. After thoroughly ruling out other differential spindle cells tumors that present S100 protein, we confirmed that this is a rare case of pancreatic desmoid-type fibromatosis presenting S100 positivity. To the best of our knowledge this is the first case of pancreatic desmoid-like fibromatosis positive for S100 protein.

Nuclear staining for β-catenin is a consistent finding in more than 80% of cases. Although it greatly aids in the diagnosis, it is not specific for this type of tumor since it is also seen in a variety of other tumors. The diagnosis can further be confirmed by screening for mutations (mainly in exon 3) of the β-catenin gene, which are found in 85% of sporadic cases [11, 13].

The prognosis is not well known but Jia *et al*. have reported a favorable outcome with 100% overall survival and > 80% disease-free survival [5]. There is, however, a relatively high incidence of postoperative recurrence rate of approximately 19 to 77% in sporadic cases. The recurrence rate can reach as high as 90% in intra-abdominal DTs associated with FAP or Gardner syndrome [5, 16].

The current first-line treatment for DT is complete resection (radical resection) with free margins. Better outcomes are achieved by performance of a resection with wide, free margins for the prevention of tumor recurrence considering the locally invasive behavior of this tumor. Experts are now recommending a wait-and-see policy (observation) especially in cases of static tumor, given the morbidity associated with surgical resection and the relatively high recurrence rate.

Adjuvant therapies such as systemic chemotherapy (vinblastine, methotrexate, doxorubicin, and dacarbazine), radiotherapy, and molecular target therapy (tamoxifen) have been proposed particularly for patients with high risk for surgery. To date however, there are no established clinical treatment guidelines for DTs. A few successes have been reported with the use of NSAIDs and celecoxib as COX2 inhibitors, but their mechanism of action is not well documented and verified [4, 5, 16, 19].

## Conclusions

The diagnosis of desmoid-type fibromatosis can be challenging because of two main factors: (1) the clinical presentation is nonspecific and thus results frequently in incidental diagnosis; and (2) there is no specific clinical investigation for this tumor. Pathological and immunohistochemical testing are important for confirmatory diagnosis, where β-catenin nuclear staining must be demonstrated.

Another important characteristic of desmoid-type fibromatosis is their expression or not of S100 protein. Most DTs are negative for S100 protein but a small percentage of cases can demonstrate S100 protein immunopositivity. This is an important diagnostic criterion that must be taken into consideration when confronting this type of tumor.

DT can present as either a solid, cystic, or solid-cystic mass and the treatment of choice remains radical surgical resection of the tumor. Adjuvant chemotherapy and/or radiotherapy are recommended for patients with high risk for surgery. In static tumors a watch and wait policy may be the best option.

## Abbreviations

*APC*: Adenomatous polyposis coli
*COX2*: Cytochrome c oxidase 2
CT: Computed tomography
DT: Desmoid tumor
FAP: Familial adenomatous polyposis
*MMP*: Matrix metallopeptidase
MRI: Magnetic resonance imaging
*PDGF*: Platelet-derived growth factor
*RB1*: Retinoblastoma 1
*RHAMM*: Hyaluronan-mediated motility receptor
SMA: Smooth muscle actin
